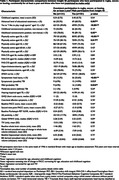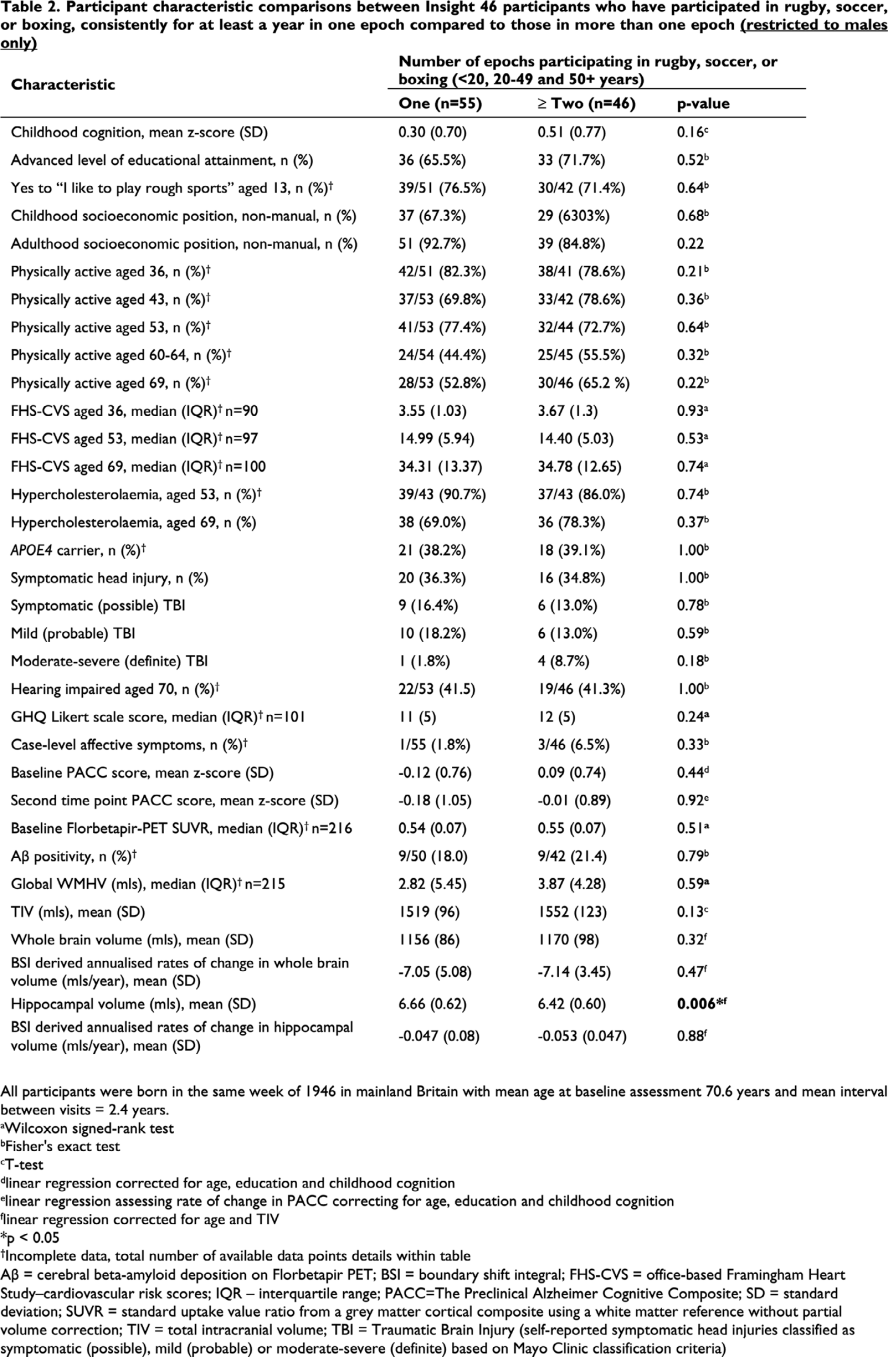# The long‐term impact of rugby, soccer and boxing participation on brain health in older adults from the general population: insights from the 1946 British birth cohort

**DOI:** 10.1002/alz.085061

**Published:** 2025-01-09

**Authors:** Thomas D Parker, Sarah‐Naomi James, Sarah E Keuss, Ashvini Keshavan, William Coath, David M Cash, Kirsty Lu, Jennifer M Nicholas, Carole H Sudre, Sebastian J Crutch, Nick C Fox, Marcus Richards, Jonathan M Schott

**Affiliations:** ^1^ UK Dementia Research Institute Centre for Care Research and Technology, London United Kingdom; ^2^ Imperial College London, Department of Brain Sciences, London United Kingdom; ^3^ Dementia Research Centre, UCL Queen Square Institute of Neurology, University College London, London United Kingdom; ^4^ MRC Unit for Lifelong Health and Ageing at UCL, London United Kingdom; ^5^ Dementia Research Centre, UCL Queen Square Institute of Neurology, London United Kingdom; ^6^ UK Dementia Research Institute at UCL, London United Kingdom; ^7^ Department of Medical Statistics, London School of Hygiene and Tropical Medicine, London United Kingdom; ^8^ Centre for Medical Image Computing, Department of Medical Physics and Biomedical Engineering, University College London, London United Kingdom; ^9^ King’s College London, London United Kingdom; ^10^ MRC Unit for Lifelong Health & Ageing at UCL, London United Kingdom

## Abstract

**Background:**

In elite athletes, participation in sports associated with repetitive head injury exposure has been linked to an increased risk of neurodegeneration later in life. However, there has been limited study in more general populations. We aimed to investigate whether participation in such sports impacted outcomes relevant to brain health in a cohort of British‐born older adults.

**Methods:**

441 participants from Insight 46 (a sub‐study of the 1946 British Birth Cohort) were asked whether they participated in rugby, soccer, or boxing, consistently for at least a year (yes/no) at various epochs of life (<20, 20‐49 and 50+ years). Individuals underwent baseline florbetapir‐PET, serial brain MRI and cognitive assessments (mean age at baseline assessment 70.6 years, mean interval between visits = 2.4 years). A range of life course data were included in analyses, including attitudes to sports, education, engagement in physical activity and self‐reported symptomatic head injury.

**Result:**

As only one female identified as participating in rugby, soccer, or boxing, only males were included in analyses: 101/232 males (43.5%) stated they participated in at least one of these sports, 46 of whom participated for more than one epoch. Univariate analyses revealed participation was associated with a range of demographic factors including: a preference “to play rough sports” aged 13 (p<0.001); a higher level of education (p = 0.007); engagement in physical activity aged 36 (p<0.001); and 43 (p = 0.017); and greater risk of self‐reported symptomatic head injury (35.3% vs 14.7%, p<0.001).

There were no significant associations between participation in these sports and baseline cognitive function or decline, beta‐amyloid deposition, white matter hypertintensity volume, or baseline/longitudinal change in total brain or hippocampal volume (table 1).

Longer duration of participation (≥ two epochs vs one) was associated with lower hippocampal volume at baseline (p = 0.006) but did not predict subsequent atrophy or any other outcome examined (table 2).

**Conclusion:**

Previous exposure to sports associated with repetitive head injuries is common amongst British‐born older adults and associated with a range of demographic factors relevant to brain health. Longer duration of exposure predicted lower hippocampal volume age 70 years, but there was no evidence of significantly accelerated atrophy or cognitive decline beyond this.